# Supplementary material for: Time-restricted feeding leads to sex- and organ-specific responses in the murine digestive system
Source: PLoS One. 2025 Sep 10;20(9):e0332295. doi: 10.1371/journal.pone.0332295 (PMC12422475; doi:10.1371/journal.pone.0332295)
Supplement: Attachment — Submitted filename: Response to reviewers.docx [file pone.0332295.s002.docx]

**Dear Reviewers and Editors,**

We appreciate your thoughtful and constructive feedback on our manuscript. Your comments have been considered, and multiple suggestions have been incorporated into the manuscript to improve its quality and objectivity. We also provide a point-by-point response to the major concerns and limitations raised in the following pages of this letter.

Additionally, we want to provide context about the circumstances of this study, specifically why conducting further experiments is not feasible. The study was funded by a 1.5-year mobility grant awarded to L. Oparija-Rogenmozere to travel to Australia and carry out the experiments at the University of Melbourne. The grant required returning to the grantor's country (Switzerland) after its completion. Unfortunately, the grant period (October 2019 - March 2021) coincided with significant COVID-19 restrictions. From March to May and July to November 2020, we had little or no access to laboratories and animal facilities, which forced us to reduce the originally planned experimental groups and conditions.

After the initial lockdowns, further long-term animal experiments were still discouraged due to the high risk of additional COVID-19 lockdowns in Melbourne (and multiple of those did happen during 2021). As a result, the available funding was redirected to other projects that were feasible under the restricted conditions. Moreover, delays in consumable deliveries and limited access to external facilities led to gene expression data being received only after the grant had ended, at which point L. Oparija-Rogenmozere had already returned to Switzerland.

Currently, almost all co-authors have left the lab where the research was conducted. Moreover, no funding, necessary equipment, or a valid animal license are available to conduct further experiments. We understand that this does not mitigate the critical concerns, but we hope that you will consider the revised and improved version of our manuscript for publication, despite our inability to provide additional data.

**Best regards,**
L. Oparija-Rogenmozere and M. R. Di Natale

**Suggestion to include relevant papers**

Reviewer 1: Reference related to food entrainment should be more balanced. It is unfair to conclude current research is fragmented (Lines 111-112). The work may benefit from including [PMID:11161204](https://www.science.org/doi/10.1126/science.291.5503.490?url_ver=Z39.88-2003&rfr_id=ori:rid:crossref.org&rfr_dat=cr_pub%20%200pubmed), [12882317](https://onlinelibrary.wiley.com/doi/full/10.1034/j.1601-183X.2003.00005.x?sid=nlm%3Apubmed), [19940241](https://pmc.ncbi.nlm.nih.gov/articles/PMC2795502/), [22608008](https://pmc.ncbi.nlm.nih.gov/articles/PMC3491655/), [32475077](https://pmc.ncbi.nlm.nih.gov/articles/PMC7496197/), [33889826](https://pubmed.ncbi.nlm.nih.gov/33889826/), [34550736](https://pubmed.ncbi.nlm.nih.gov/34550736/), [35032416](https://pubmed.ncbi.nlm.nih.gov/35032416/), [37267101](https://pubmed.ncbi.nlm.nih.gov/37267101/), [37773240](https://pubmed.ncbi.nlm.nih.gov/37773240/), [40088888](https://pubmed.ncbi.nlm.nih.gov/40088888/)

Response: We thank the reviewer for suggesting additional references and fully agree that the field of food entrainment has indeed made substantial progress in recent years. We have reviewed the suggested literature and have incorporated several of these references to provide a more balanced overview of the field.

That said, we respectfully stand by our original statement that **research on food entrainment, particularly in peripheral organs, remains fragmented**. This view is based on for several reasons:

1. **Narrow scope**: most studies still focus on a single organ, sex, gene groups, or entrainment regime. Comprehensive studies examining how **multiple tissues adapt to food intake time in an organ-, sex-, and time-restricted feeding (TRF) duration-specific manner** are still uncommon, despite the known complexity of systemic adaptation to feeding cues. We are aware that certain ethical and space constraints make such large-scale studies difficult to perform; however, they would be essential in providing an overview of systemic adaptation to food intake.
2. **Digestive organs remain understudied**: research on food entrainment in peripheral tissues has disproportionately focused on the liver (with good representation of muscle and adipose tissue), while other key digestive tissues, such as **stomach (often only studied in context of ghrelin rhythm) and the upper GI tract (especially when it comes to distinction of duodenal, jejunal and ileal responses)** are still understudied, especially at *in vivo* conditions. Yet, these tissues are the first to interact with ingested food and thus should not be excluded from the food-anticipatory physiology.
3. ***Ex vivo* approach:** some studies that attempt to characterize food entrainment in the digestive system use tissue explant cultures (e.g., PMID: **12882317**). While this model is convenient for assessing a single gene expression rhythm, it does not capture *in vivo* dynamics or systemic cues influencing food entrainment (e.g., hormonal, neural, or environmental) or changes in other parameters (metabolic genes or phenotype). Therefore, such models are great in providing mechanistic insights and measuring free-running rhythms, but fail to represent the consequences of *in vivo* food entrainment.

Revision to manuscript: In addition to including relevant references in Introduction, Results and Discussion, we have revised our statements in the **Introduction** to clarify that **while substantial progress has been made**, the field still lacks a cohesive and integrative view of food entrainment across peripheral organs, sexes, and time scales. Such clarification acknowledges important contributions of prior work and yet stresses the remaining knowledge gaps, some of which our study sought to address.

**Limitation #1: lack of circadian sampling.**

Reviewer #1:  The main assessment is based on gene expression profiling at three time points related to food intake. The rationale for this design should be clarified. In contrast, the main experimental paradigm for assessing food entrainment in peripheral tissues is a high-resolution sampling scheme throughout the day (every 3-4 h for 1-2 days). While the findings related to organ-specific and sex-specific gene expression are interesting, these findings are not interpretable concerning the adaptation rates to meal time (Line 51). The authors may reframe the conclusions and explain their rationales and the difference with existing studies in the last section of Intro or in the first section of Results.

Reviewer #2: Lack of Circadian Sampling. While the manuscript discusses clock gene expression and clock-regulated genes (e.g., Cck, Leptin), these assessments were conducted at a single time point. Since circadian gene expression is inherently rhythmic, multiple sampling points over 24 hours are required to determine rhythmicity, amplitude, and phase. The current approach does not support claims related to circadian regulation. A temporal sampling strategy would significantly strengthen the chronobiological component of this work.

Response: We thank the reviewers for their thoughtful comments regarding circadian sampling. We agree that a classical circadian analysis typically involves high-resolution sampling, which is essential for assessing endogenous rhythmicity, amplitude, and phase. However, our study was designed with a different objective - to investigate how time-restricted feeding (TRF) influences gene expression across peripheral tissues in a biologically and behaviorally relevant context. For this objective, we selected three strategic timepoints within the 24-hour cycle: food anticipation, intake, and postprandial period, rather than conducting a full circadian time course. These timepoints were chosen not to track endogenous circadian oscillations per se, but to capture key physiological windows associated with food-driven responses, which are central to our aims.

Importantly, the design allowed us to align equivalent physiological states across different TRF regimes within the same Zeitgeber Time (ZT). For example, the food anticipation timepoint (A) for night-fed (NF) mice corresponds in ZT to the postprandial timepoint (P) in day-fed (DF) mice and vice versa (see Fig. 1). This strategic alignment enabled us to distinguish food-driven and light-driven changes, despite the limited sampling. For instance, as shown in Figure 3, Bmal1 expression in the duodenum on Day 3 is consistently higher at the postprandial timepoint across all TRF regimes, even though these occur at different ZTs. This suggests that gene expression patterns are entrained to feeding rather than the LD cycle, which is a hallmark of food-entrainment. We would like to clarify that our study does not aim to assess full circadian rhythmicity or clock amplitude. Rather, our focus is on food-entrainment and food-driven regulation, which are related to but distinct from endogenous circadian control. Time-restricted feeding is a well-established entrainment cue for peripheral clocks (Mistlberger, 2011, *Physiol Behav*, PMID: 21527266), and our study adds novel insight into how quickly and robustly peripheral gene expression adapts to new feeding schedules, with attention to organ- and sex-specific dynamics.

We appreciate the suggestion to clarify the distinction between circadian and food-driven regulation, and agree that our original introduction may have placed undue emphasis on circadian mechanisms. In response, we have revised our manuscript to better reflect the primary focus of our work: food-driven responses under TRF, rather than assessment of endogenous circadian rhythms.

Unfortunately, we are unable to perform extended sampling or new experiments for this project. The study was conducted under a limited funding period (1.5 years), the animal license has since expired, and most contributing authors, including the two first co-authors, have left the lab. Nevertheless, we believe that the current dataset offers valuable and underexplored insights into TRF-induced shifts in gene expression, especially in terms of baseline gene expression changes (e.g., overall up- or down-regulation under specific TRF) and cross-regime comparisons.

**Revision to manuscript: We have adjusted the manuscript to shift focus from circadian rhythms to food-driven regulation.** We have also included this limitation in the **Discussion**: “A substantial limitation of our study is the absence of high-resolution circadian sampling, preventing an assessment and characterization of rhythmic parameters such as amplitude and phase. While this design choice limits conclusions about endogenous circadian regulation, our goal was to focus on food-driven responses under TRF, using three biologically relevant timepoints aligned to key feeding-related states (anticipation, intake, and postprandial period). A strategic alignment of these states across different TRF regimes to shared ZTs allowed us to distinguish feeding-driven gene expression from light-driven circadian cues, even with limited sampling. While this approach does not substitute for full circadian profiling, it was successful in providing novel insights into the adaptation of peripheral gene expression to feeding schedules *in vivo*.”

**Limitation #2: lack of oestrous cycle monitoring.**

Reviewer #2**:** Although the manuscript investigates sex differences, it does not address or control for the estrous cycle in female mice. Hormonal fluctuations across the estrous cycle can significantly affect metabolism, gene expression, and behavior. This is particularly relevant for parameters such as glucose tolerance. Without estrous cycle monitoring (e.g., via vaginal cytology), the interpretation of female data is limited, and variability may obscure true sex differences. If monitoring was not performed, this limitation should at least be acknowledged and discussed.

Response: We thank the reviewer for raising this important point. Vaginal cytology was indeed not performed in this study. However, we would like to note that current literature does not consistently support the notion that female mice exhibit greater trait variability due to the estrous cycle when compared to males. For example, studies have shown that while the estrous cycle influences the **amplitude** of certain eating behaviors, it does not affect their **phase** (Alvord & Pendergast, 2024, J Biol Rhythms, PMID: 39082411). Moreover, female mice do not exhibit higher variability in food intake than males (Smarr et al., 2019, PLoS One, PMID: 31306437). In the context of glucose metabolism, Kennard et al. (2022, Diabetes Obes Metab, PMID: 35815375) reported that male mice exhibit greater variability in blood glucose levels than females, regardless of estrous cycle stage, fasting protocol, or cage-change method. Similarly, the review by Mauvais-Jarvis et al. (2017, Cell Metab, PMID: 28591630) concludes that estrous cycle effects on glucose homeostasis are minimal.

Furthermore, a large meta-analysis comparing male and female variability across numerous behavioral, physiological, and molecular traits found no evidence for greater variability in females, instead concluding that for some traits, males actually exhibited more variability (Prendergast et al., 2014, Neurosci Biobehav Rev, PMID: 24456941). In circadian studies specifically, males have shown higher intra-individual variability in core body temperature and similar variability in locomotor activity compared to females (Smarr et al., 2017, Biol Sex Differ, PMID: 28203366). Additionally, transcriptomic studies examining food entrainment in female mice have demonstrated robust sex differences in gene expression without controlling for estrous stage (e.g., Xin et al., 2021, iScience, PMID: 33889826).

Taken together, these findings suggest that while the estrous cycle is a biologically relevant factor, its impact on overall physiological variability in female mice may be limited and does not necessarily obscure the detection of sex differences. Nonetheless, we agree that estrous cycle monitoring can be informative in certain contexts and now acknowledge its absence in our study.

**Revision to manuscript:** We have included the following statement in the **Discussion, under subsection Limitations of the study**: “Estrous cycle staging was not performed in this study due to logistical constraints. However, previous studies have shown that female mice do not exhibit greater variability than males in physiological, behavioural, or molecular traits, suggesting that estrous cycle monitoring may not be necessary for detecting robust sex-specific differences in these contexts”.

**Limitation #3. Cage Replacement and Stress.**

Reviewer #2: The Methods state that mice were group-housed and placed into new cages daily during food restriction to prevent feeding from spillage. Daily cage changes are known to induce stress, particularly in male mice, due to the re-establishment of social hierarchies. This stress response can influence metabolism and behavior and may differ by sex. The authors should consider and discuss the potential impact of this variable on the outcomes.

Response: We appreciate the reviewer’s comment and agree that cage changing can induce stress, particularly in male mice, and that this may influence metabolic and behavioral outcomes. In our study, although mice were transferred daily between designated “food” and “empty” cages during TRF, the cages remained consistent for a full week and were only changed once per week (with the “empty” cage becoming the new “food” cage, and a new cage becoming the “empty” cage) as a part of routine cleaning. To mitigate potential stress, portions of nesting material (e.g., tissues) and enrichment objects (e.g., shelters) were transferred along with the mice, while the soiled bedding was not reused. This approach has been shown to reduce stress responses and minimize male aggression (Van Loo et al., 2000, Animal Welfare, DOI: 10.1017/S0962728600022752).

All experimental groups were subjected to the same handling routine, and we did not observe signs of elevated distress, such as excessive aggression, injury, or unexplained weight changes. While we acknowledge that daily handling may have induced a mild stress response (Rasmussen et al., 2011, J Am Assoc Lab Anim Sci, PMID: 21838975), we considered the potential impact of food spillage on intake measurements to be a greater confounding factor. Previous studies have shown that mice at similar ages drop approximately 1 g of food per mouse per day (Starr & Saito, 2012, J Gerontol A Biol Sci Med Sci, PMID: 22451471), which could substantially skew food intake-based parameters. However, we have made adjustments to the manuscript to ensure this limitation is addressed.

Revision to manuscript: We have addressed this potential tradeoff as a methodological limitation in the **Discussion**: “A potential limitation of our study is the daily cage transfer during the TRF, which could have introduced mild handling-related stress, particularly in male mice, due to the re-establishment of social hierarchies. While elevated corticosterone levels and anxiety-like behaviours have been reported with frequent cage changes, efforts were made to minimize stress by using consistent “food” and “empty” cages throughout the week and by transferring portions of nesting material and enrichment objects. We prioritized this approach to reduce variability in food intake measurements caused by food spillage from the designated hopper, which can be substantial in young mice. Nonetheless, we acknowledge that stress responses may have influenced certain physiological outcomes and should be considered when interpreting the results.”

**Limitation #4. Ambient Temperature Not Reported.**

Reviewer #2: Ambient temperature was not reported in the manuscript. This is a critical oversight, especially in metabolic and circadian studies. Standard animal facility temperatures (21–23 °C) are below thermoneutrality for mice (~30 °C), leading to increased energy expenditure for thermoregulation. Importantly, thermoregulatory demands and strategies can differ between sexes, potentially contributing to the observed metabolic responses. The authors should report the housing temperature and discuss how it may have influenced their findings.

**Response:** We appreciate the reviewer’s important point regarding the influence of ambient temperature on metabolic and circadian physiology. In our study, mice were housed at standard facility conditions (22 ± 1 °C, 55-60% humidity), which are indeed below thermoneutrality. We agree that sub-thermoneutral temperatures can influence energy expenditure and potentially contribute to sex-specific differences in metabolic responses. It is also worth noting that thermoneutrality in mice can vary between the light and dark phases by approx. 4 °C, as reported by Skop et al. (2020, Cell Reports, PMID: 32294435), making strict thermoneutral housing across the entire circadian cycle hard to achieve in standard laboratory settings.

**Revision to manuscript:** We have added temperature and humidity information to the **Methods** and addressed the implications in the **Discussion**: “Mice in this study were housed at standard laboratory conditions (22 ± 1 °C, 55-60% humidity). While these temperatures are common in research settings, they are below the thermoneutral zone for mice, which may influence metabolic homeostasis and the regulation of gene expression rhythms in a sex-specific manner. However, thermoneutrality has been reported to vary between the light and dark phases by approximately 4 °C, implying that achieving consistent thermoneutral conditions throughout the LD cycle would require dynamic temperature regulation.”

General comment on limitations #2-4: The primary aim of our study was to examine how male and female mice adjust to specific time-restricted feeding schedules under identical environmental conditions. Factors such as ambient temperature, cage changes, and hormonal fluctuations may indeed differentially affect metabolism in each sex. Still, rather than confounding our findings, these variables are inherently part of the sex-specific responses we aimed to observe. Since the study was not designed to dissect mechanistic pathways, we argue that in this specific case, it is not essential to isolate each contributing factor. Instead, our findings underscore that when placed under the same environmental and feeding conditions, males and females exhibit distinct physiological and transcriptional responses, highlighting the overall sex-specific adaptation to food entrainment.

**Group Housing and Feeding Behaviour**

Reviewer #2: How many mice were housed per cage? Group housing can affect individual feeding behavior, access to food, and timing of consumption, factors that may influence metabolic outcomes. This is especially relevant in TRF protocols. The information of diet type, which contains particular energy should be mentioned in the method section.

**Response:** We thank the reviewer for this important observation. Mice were housed in groups of five per cage, per sex and feeding condition. While group housing can indeed influence feeding behaviour (Bailoo et al., 2018, *Sci Rep*, DOI: 10.1038/s41598-017-18493-6 and Whittaker et al., 2012, *Lab Anim*, PMID:2 2114068), individual housing (e.g., in metabolic cages, for precise food intake measurements) can also induce stress in a time-specific manner (Hebda-Bauer et al., 2020, *Horm Behav*, PMID: 31220462) and affect the metabolism (Ziegler et al., 2022, *J Am Assoc Lab Anim Sci*, PMID: **34996529)**. Thus, we opted for group housing to minimize prolonged isolation stress. As noted in the Discussion, measurement of individual food intake was not possible due to group housing, which we acknowledged as a limitation.

Regarding diet, all animals were fed standard Barastoc 102108 chow, as noted in the Methods.

**Revision to manuscript:** We have clarified group size and energy content of the diet in the **Methods, subsection on Experimental animal origin and housing.**

**Statistical Analysis and Data Distribution**

Reviewer #2: The manuscript states that parametric tests (e.g., t-tests, ANOVA) were used, but does not mention whether data were tested for normal distribution. Confirmation that assumptions for parametric testing were met is necessary to validate the statistical approach. Without this, the reliability of reported p-values may be questioned.

Response: We thank the reviewer for raising this concern. We did not test the data for normality, as common normality tests like Shapiro-Wilk or Kolmogorov-Smirnov tend to fail to detect non-normal distributions with small sample sizes (Razali et al., 2011, *J Stat Model Analytics*, Corpus ID: 18639594). Therefore, we used two-way ANOVA tests to compare parameters across multiple days and timepoints, and non-parametric tests (Kruskal-Wallis test with Dunn’s multiple comparison test) to identify differences between the three timepoints on a single sacrifice day within the same TRF condition. No t-tests were used in our study. Parametric tests are robust when group sizes and variances are similar (conditions met in our study). It has been shown that when normality test is applied to raw data, the choice of parametric vs non-parametric test results in only a small difference in power (Midway & White, 2025, *R Soc Open Sci*, PMID: 40309180) thus even if data show mild non-normality, tests like ANOVA, which maintain type I error probability, are still able to detect differences between the groups. Given the design and distribution of our data, we believe the use of chosen tests was justified.

Revision to manuscript: We have clarified the relevant **Methods** subsection for transparency.

**Control Group**

Reviewer 2: The authors state that a control group with ad libitum feeding was included in the study; however, explicit data from this group are not presented in the figures or adequately discussed in the text. Specifically, body weight changes and gene expression profiles appear to be shown only for the food-restricted groups subjected to different time-restricted feeding (TRF) schedules. While pre-TRF baseline weights are used for normalization, dedicated control group data are notably absent. This omission limits the interpretation of the observed effects, particularly since food restriction in this study alters the sleep–wake rhythm, a factor that can independently influence both body weight and gene expression.

Response: There is no mention or claim of the inclusion of *ad libitum* fed control group in the manuscript. All animals were subjected to an initial acclimatization period with *ad libitum* access to food, but then placed on a time-restricted feeding (TRF) protocol. Only water was available *ad libitum* during the TRF.

Instead, the **night-fed (NF)** group served as our **reference group bec**ause this feeding schedule most closely resembles the natural feeding behavior of mice (Xie et al., 2020, *BMC Biology*, PMID: 33158435). This allowed us to evaluate the impact of shifting the feeding window away from the biologically preferred period. We acknowledge that an *ad libitum* group would be necessary to investigate endogenous circadian rhythms. However, our study was not designed to assess circadian phase or amplitude. Instead, we aimed to explore how changes in food intake timing affect metabolic and transcriptional outcomes across sexes and organs under controlled environmental conditions. If a specific TRF schedule alters activity or other physiological rhythms, such as the sleep-wake cycle, with further effects on metabolic homeostasis, we assume that these are all consequences of that particular TRF for the purpose of this study. We do not consider disruptions that would not happen without TRF to be truly independent influences in our experimental setup. Therefore, while we agree that this distinction could be more clearly explained, the current design aligns with the goals of our study.

**Revision to manuscript:** To avoid confusion, we have now clarified in the **Methods** and **Discussion** that no *ad libitum* group was included, and that NF males served as the control group for all comparisons.

**Sample Size and Replication**

Reviewer # 2: The sample size reported per time point was n = 5 per sex. This may be underpowered to detect subtle but biologically meaningful differences in gene expression or physiology, especially given variability in circadian and metabolic data. Additionally, details regarding biological replicates and experimental repeats are insufficient. The authors should clarify how many independent experiments were performed and discuss power considerations.

**Response:** Each TRF condition included biological replicates of n = 5 per sex per timepoint across three independent sacrifice days, as detailed in the Methods. While the sample size per timepoint is modest and was limited by ethical and space constraints, post hoc tests (effect size/Cohen’s F) suggest that we did have enough power to detect large effects (q>3.9) and sufficient power to detect moderate or small effects (3.9>q>2). However, for detecting even smaller effects (q<2), our chosen tests could have been underpowered and could indeed benefit from larger group sizes.

We performed 2 independent experiments (mixed TRF group allocation), largely due to Covid-19 pandemic restrictions and housing space limitations that did not allow us to process all mice at once. The start of TRF for some of the mouse groups belonging to the same experiment was delayed by 1-2 days to avoid overlapping sacrifice timepoints. Sample processing for RNA extraction and DNA synthesis was performed after both experimental sets were sacrificed, across multiple days in small batches, randomly allocating samples to be co-processed on the same day. We did not use technical replicates to determine gene expression, as each sample underwent quality control (by measuring DNA concentration and contamination, and an expression level of a housekeeping gene) before it was used for Dynamic Array IFC (Fluidigm system). All samples from the same organ were randomly allocated between two Fluidigm plates (24 genes x 192 samples), with a few samples loaded on both plates to detect any plate-specific differences.

**Revision to manuscript:** We have included more details on the experimental sets and group sizes in the respective subsections in the **Methods** (Food entrainment of mice) and addressed this limitation in the **Discussion**: “We used 5 mice per timepoint per sacrifice day per sex. Although the chosen sample size was small, post hoc tests (effect size/Cohen’s F) for randomly chosen parameters revealed enough power to detect large effects (q>3.9) and sufficient power to detect moderate effects (3.9>q>2). However, to detect small effects (q<2), our chosen statistical tests could have been underpowered, suggesting that our study could have benefited from larger group sizes.”

**Specific Comments**

Abstract (lines 32–43): This section lacks clarity in distinguishing findings by sex. It is unclear which effects were observed in males versus females. The authors should revise this passage to specify sex-specific outcomes more clearly.

Revision to manuscript: We have revised the abstract to specify sex-specific differences.

Glucose Tolerance Tests and Estrous Cycle: The researchers didn't say whether they controlled for or noted the reproductive cycle phase of female subjects during the testing, which could be a methodological oversight. Since estrogen and progesterone modulate glucose metabolism, unaccounted hormonal variation could affect these outcomes. Without controlling for estrous cycle phase, sex comparisons in glucose tolerance should be interpreted with caution.

Response: Please see the response regarding this concern under Limitation #2.
